# Increased risk of aspirin-induced gastric mucosal erosion in elderly Chinese men harboring SLCO1B1*1b/*1b while using aspirin and an ACEI or ARB concomitantly

**DOI:** 10.1186/s12881-019-0918-4

**Published:** 2019-11-14

**Authors:** Lei Duan, Yongyi Bai, Man Li, Huiying Li, Yanping Li, Hongbin Liu

**Affiliations:** 1 Medical School of Chinese PLA, Beijing, 100853 China; 2 Department of Geriatric Cardiology, National Clinical Research Center for Geriatric Diseases, the Second Medical Center of PLA General Hospital, 28 Fuxing Road, Beijing, 100853 China

**Keywords:** Single nucleotide polymorphism, Aspirin, Gastric mucosa erosion, *SLCO1B1*, Pharmacogenomics

## Abstract

**Background:**

It is well established that long-term use of aspirin can cause gastric mucosal injury. ACEIs and ARBs are inversely related to gastric ulcer development. This study aimed to evaluate the relationship between *SLCO1B1* polymorphisms, which can affect ACEI and ARB transport, and gastric mucosal erosion in elderly male Chinese patients with cardiovascular disease who use aspirin.

**Methods:**

Patients taking aspirin and an ACEI or ARB concomitantly who had undergone endoscopic screening for gastric erosion were analyzed for *SLCO1B1* polymorphisms by a TaqMan assay.

**Results:**

The frequency of the SLCO1B1*1b/*1b diplotype (42% vs. 24%; *p* = 0.002) was significantly higher in the gastric mucosal erosion group than in the control group. After adjustment for significant factors, SLCO1B1*1b/*1b (OR, 2.64; 95% CI, 1.59–4.17; *p* < 0.05) was found to be associated with gastric mucosal erosion in aspirin users.

**Conclusions:**

The presence of the SLCO1B1*1b/*1b diplotype may be a risk factor for aspirin-induced gastric mucosal erosion in elderly Chinese men taking aspirin and an ACEI or ARB concomitantly.

## Introduction

Cardiovascular disease is the leading cause of disability and death in elderly Chinese males. Aspirin is a common antiplatelet drug used to prevent and treat ischemic vascular disease. However, antiplatelet therapy results in a higher incidence of gastrointestinal (GI) injury, particularly gastric mucosal ulcers and hemorrhage [1]. GI injury is related to a poor prognosis of cardiovascular disease [2]. Moreover, the mechanisms and genetic influence of antiplatelet-related GI injury remain largely unknown.

It is well established that long-term use of aspirin may result in gastric mucosal injury but that concomitant use of aspirin and an angiotensin-converting enzyme inhibitor (ACEI) or angiotensin II AT-1 receptor blocker (ARB) may attenuate aspirin-induced gastric injury [3]. Their transport from circulating blood to hepatocytes requires the uptake transporter organic anion transporting polypeptide 1B1 (OATP1B1), which is encoded by the solute carrier organic anion transporter 1B1 (*SLCO1B1*) gene [4].

Elderly patients are the major demographic using aspirin; thus, identifying the risk factors of early-phase gastric mucosal injury such as gastric mucosal erosion, which can evolve into gastric ulcers but is confined to the gastric mucosa (has not reached the mucosal muscularis), is critical for preventing aspirin side effects. The purpose of this study was to explore the genetic and clinical risk factors associated with gastric mucosal erosion in elderly male Chinese patients with cardiovascular disease taking aspirin and an ACEI or ARB concomitantly.

## Materials and methods

### Study population

This case–control study analyzed 268 elderly male Chinese patients ≥60 years old who took 100 mg of aspirin daily between 2013 and 2018 for ischemic cardiovascular disease and were undergoing annual upper GI endoscopy persistently, including 143 men in the case group and 125 men in the control group according the gastroscope inspection results, at the Second Medical Center of People’s Liberation Army of China (PLA) General Hospital. All participants were also administered ACEIs or ARBs for hypertension. All the endoscopy results of patients were not abnormal prior to 2013, and endoscopy screening findings were stable during antiplatelet therapy. Patients were excluded if they had gastric cancer or other malignant lesions or took other antiplatelet and anticoagulant drugs. This study was approved by the ethics committee of PLA General Hospital. All participants provided written informed consent.

### Clinical data collection

Information on the participants was collected from physical examinations and laboratory studies. The physical examination data extracted from the hospital records were age, body mass index, systolic and diastolic blood pressure, current cigarette smoking, alcohol consumption, *Helicobacter pylori* status, history of gastric ulcer, diabetes mellitus, hyperlipidemia, ischemic cerebrovascular disease, and use of proton-pump inhibitors (PPIs), nonsteroidal anti-inflammatory drugs (NSAIDs), ARBs or ACEIs and statins. *H. pylori* status was detected using the C^13^ urea breath test. Diabetes was diagnosed when the subject had a fasting glucose ≥7.0 mmol/L or ≥ 11.1 mmol/L 2 h after oral glucose challenge. Gastric mucosal erosion was defined as erosion injury visible on endoscopy that was limited to the mucosal layer. Hyperlipidemia was defined as low-density lipoprotein cholesterol (LDL-C) ≥3.64 mmol/L or high-density lipoprotein cholesterol (HDL-C) ≤0.91 mmol/L or total triglyceride (TG) ≥1.7 mmol/L or total cholesterol (TC) ≥5.72 mmol/L. Cerebrovascular disease was defined as a previous ischemic stroke based on hospital records. The clinical data were recorded by physicians at the Department of Geriatric Cardiology of PLA General Hospital who were trained by the research team.

### Laboratory measurements

Blood samples were collected in prechilled vacutainers after at least 12 h of overnight fasting. The activated partial thromboplastin time (APTT) was determined using an automatic coagulometer (SYSMAX CA-1500, Sysmex Shanghai Ltd., Japan), the platelet count was determined using an automatic hematology analyzer (Nihon Kohden MEK-7222 K, Japan), and TC, LDL-C, glucose, and serum creatinine (SCr) values were determined using an automatic biochemical analyzer (Hitachi 7400, Japan). All testing was performed in the same laboratory by trained personnel following the criteria of the World Health Organization Lipid Reference Laboratories. Renal function was assessed by the estimated glomerular filtration rate (eGFR), which was calculated by the following formula: eGFR (ml/min/1.73 m^2^) = 175 × standard SCr (mg/dl) – 1.234 × age – 0.179. [standard SCr (mg/dl) = SCr (mg/dl) (detected by an enzymatic method) × 0.795 + 0.29] [5, 6].

### DNA isolation and genotyping

Two hundred microliters of venous blood from every patient was extracted and collected into an EDTA anticoagulant tube (Biotend, Shanghai, China). The blood genomic DNA isolation kit (DP318, TIANGEN Biotech, Beijing, China) was used to extract DNA samples, and a Q3000 ultraviolet spectrophotometer (Quawell, San Jose, CA, USA) was employed to detect the concentration of the DNA samples. The genotypes were determined using a TaqMan assay and an ABI Prism Sequence Detector 7000 (Applied Biosystems, Foster City, Calif) according to standard protocols. Genotyping for *SLCO1B A388G* (rs2306283) (forward primer 5′-TGTTGGTTTTATTGACGGAAGC-3′, reverse primer 5′-CCCACTATCTCAGGTGATGCTCTA-3′) and *SLCO1B T521C* (rs4149056) (forward primer 5′-TCAACATCGACCTTATCCACTTG-3′, reverse primer 5′-CAATGTAAGAAAGCCCCAATGG-3′) was performed for each sample.

### Statistical analysis

The distribution of continuous variables was tested for normality using the one-sample Kolmogorov-Smirnov test. Data were presented as numbers and frequencies for categorical variables and as means ± standard deviation (SD) for continuous variables. Baseline characteristics and SNP genotypes were compared with the chi-square test for categorical variables and unpaired Student’s *t*-test for continuous variables. The odds ratio (OR) and 95% confidence interval (CI) were obtained by Mantel–Haenszel statistics and multiple logistic regression analysis to identify the risk or preventive factors after adjustment for the other significant factors determined by univariate analysis. Differences in the genotype frequencies between the two groups and in Hardy–Weinberg equilibrium of allele frequencies at individual loci by comparing the observed and expected genotype frequencies were assessed using the Chi-squared test or Fisher’s exact probability test. All reported *p*-values were two-tailed, and *p* < 0.05 was considered statistically significant. Analyses were performed using SPSS software version 19.0 (SPSS IBM Corporation, Armonk, NY, USA).

## Results

### Subject characteristics

We enrolled 268 male patients (62–89 years old; average age: 75.2 years). Among these patients, 143 had gastric mucosal erosion, and 125 had normal gastric mucosa. The baseline clinical characteristics according to the endoscopy results are presented in Table 1. Age (78.5 ± 10.1 vs. 71.5 ± 9.6 years, *p* < 0.001), SCr plasma levels (96 ± 35.3 vs. 88.3 ± 45.8 μmol/ L, *p* < 0.01), history of gastric ulcer (*n* = 20, 14% vs. *n* = 7, 5.6%, *p* = 0.02) and co-existing diabetes (*n* = 58, 40.6% vs. *n* = 30, 24%, *p* < 0.01) were found to be significantly higher in the gastric erosion group than in the control group. However, eGFR (55.8 ± 23.1 vs. 70.9 ± 18.5 ml/min/1.73 m^2^, *p* > 0.001) and PPI use (*n* = 26, 18.2% vs. *n* = 63, 50.4%, *p* < 0.001) were lower in the gastric erosion group than in the control group. These baseline clinical factors contributed to aspirin-induced gastric mucosal erosion to a significantly greater extent in the univariate analysis.

**Table 1:** Demographic and clinical characteristics of the study patients

Variable	Control *n* = 125	Case *n* = 143	*p*
Age, yrs	71.5 (9.6)	78.5 (10.1)	<0.001
Body mass index (kg/m^2^)	24.5 (2.6)	25 (2.9)	0.79
SBP (mmHg)	126.5 (14.7)	131.3 (14.4)	0.23
DBP (mmHg)	73.4 (9)	71.7 (9.9)	0.13
Glucose (mmol/L)	6.6 (2.3)	6.9 (2.7)	0.33
TC (mmol/L)	4.3 (0.9)	4.2 (0.8)	0.51
LDL-C (mmol/L)	3.0 (0.9)	3.1 (0.6)	0.93
APTT (s)	35.2 (3.8)	39.6 (2.6)	0.15
Platelet count (10^9^ cells/L)	261.6 (43.3)	239.2 (40.5)	0.08
Creatinine (μmol/ L)	88.3 (45.8)	96 (35.3)	<0.01
eGFR (ml/min/1.73 m^2^)	70.9 (18.5)	55.8 (23.1)	<0.001
Alcohol drinking, n (%)	8 (6.4)	10 (7.0)	0.85
Smoking, n (%)	18 (14.4)	25 (17.5)	0.49
*H. pylori-*positive, n (%)	18 (14.4)	30 (20.9)	0.16
History of gastric ulcers, n (%)	7 (5.6)	20 (14)	0.02
Diabetes mellitus, n (%)	30 (24)	58 (40.6)	<0.01
Hyperlipidemia, n (%)	58 (46.4)	68 (47.6)	0.85
Cerebrovascular disease, n (%)	32 (25.6)	31 (21.7)	0.45
ARBs or ACEIs, n (%)	125 (100)	143 (100)	1.0
Statins, n (%)	44 (35.2)	59 (41.3)	0.31
PPIs, n (%)	63 (50.4)	26 (18.2)	<0.001
NSAIDs, n (%)	2 (1.6)	7 (4.9)	0.18

Data are presented as the mean ± SD, or n (%), as appropriate

Abbreviations: *ACEIs* Angiotensin-converting enzyme inhibitors, *APTT* Activated partial thromboplastin time, *ARBs* Angiotensin receptor blockers, *DBP* Diastolic blood pressure, *eGFR* Estimated glomerular filtration rate, *LDL-C* Low-density lipoprotein cholesterol, *NSAIDs* Nonsteroidal anti-inflammatory drug, *PPIs* Proton-pump inhibitors, *SBP* Systolic blood pressure, *TC* Total cholesterol

### *SLCO1B* genotype distribution

All subjects were successfully genotyped. The allele frequencies of the polymorphisms did not deviate significantly from those expected under Hardy–Weinberg equilibrium. The frequencies of the T allele (85.7% vs. 72.0%, *p* < 0.001) and the *SLCO1B1 521TT* genotype (77.6% vs. 58.4%, *p* = 0.001) were significantly higher in the gastric erosion group than in the control group. Accordingly, the frequencies of the *SLCO1B1 521TC* genotype (16.1% vs. 27.2%, *p* = 0.027) and *SLCO1B1 521CC* genotype (6.4% vs. 14.4%, *p* = 0.028) were significantly lower in the gastric erosion group than in the control group (Table 2).

**Table 2:** Allele and genotype frequencies of *SLCO1B1* in patients taking aspirin

Gene	Allele frequencies *p*† value for HWE	Control *n* = 250(%)	Case *n* = 286 (%)	p	Genotype	Control *n* = 125 (%)	Case *n* = 143 (%)	*p*
*SLCO1B1*	A = 0.28	78 (31.2)	74 (25.9)	0.172	*AA*	9 (7.2)	10 (7.0)	0.947
*388 A > G*	G = 0.72	172 (68.8)	212 (74.1)		*AG*	60 (48)	54 (37.8)	0.091
*rs2306283*	P† = 0.44				*GG*	56 (44.8)	79 (55.2)	0.088
*SLCO1B1*	T = 0.79	180 (72.0)	245 (85.7)	<0.001	*TT*	73 (58.4)	111 (77.6)	0.001
*521 T > C*	C = 0.21	70 (28.0)	41 (14.3)		*TC*	34 (27.2)	23 (16.1)	0.027
*rs4149056*	P† = 0.37				*CC*	18 (14.4)	9 (6.3)	0.028

*p* values from the Chi-squared test. †, Hardy–Weinberg equilibrium (HWE) of allele frequencies at individual loci was assessed by comparing the observed and expected genotype frequencies

The frequencies of the SLCO1B1*1b haplotype (62.6% vs. 44.8%, *p* < 0.001) and SLCO1B1*1b/*1b diplotype (42.0% vs. 24.0%, *p* = 0.002) were significantly higher in the gastric erosion group than in the control group. The frequencies of the SLCO1B1*15 haplotype (11.5% vs. 24.0%, *p* < 0.001), SLCO1B1*15/*15 diplotype (0.7% vs. 6.4%, *p* = 0.014) and SLCO1B1*1a/*15 diplotype (3.5% vs. 12.8%, *p* = 0.005) were significantly lower in the gastric erosion group than in the control group (Tables 3 and 4).

**Table 3:** Frequencies of the haplotypes of *SLCO1B1* in patients taking aspirin

Haplotype	Allele of 388/521	Control *n* = 250 (%)	Case *n* = 286 (%)	*p*
*1a	*A/T*	68 (27.2)	66 (23.1)	0.271
*1b	*G/T*	112 (44.8)	179 (62.6)	<0.001
*5	*A/C*	10 (4.0)	8 (2.8)	0.441
*15	*G/C*	60 (24.0)	33 (11.5)	<0.001

*p* values from the Chi-squared test

**Table 4:** Frequencies of the diplotypes of *SLCO1B1* in patients taking aspirin

Diplotype	Allele of 388–521/ 388–521	Control *n* = 125 (%)	Case *n* = 143 (%)	*p*
*1a/*1a	*AT/AT*	9 (7.2)	10 (7)	0.947
*1b/*1b	*GT/GT*	30 (24.0)	60 (42.0)	0.002
*15/*15	*GC/GC*	8 (6.4)	1 (0.7)	0.014
*1a/*1b	*AT/GT*	34 (27.2)	41 (28.7)	0.789
*1a/*15	*AC/GT*	16 (12.8)	5 (3.5)	0.005
*1b/*15	*GC/GT*	18 (14.4)	18 (12.6)	0.664
*5/*15	*AC/GC*	10 (8)	8 (5.6)	0.433

*p* values from the Chi-squared test

### Factors associated with gastric mucosal erosion

After adjusting for significant factors in the univariate analysis, older age (OR 1.33, 95% CI 1.04–2.87, *p* < 0.05), lower eGFR level (8.04, 3.02–22.6, *p* < 0.01), history of gastric ulcer (2.41, 1.08–5.01, *p* < 0.05), decreased use of a PPI (0.18, 0.11–0.31, *p* < 0.001) and the SLCO1B1*1b/*1b diplotype (2.64, 1.59–4.17, *p* < 0.05) were significantly associated with gastric mucosal erosion in multiple logistic regression analysis (Table 5).

**Table 5:** Association between various related factors and gastric mucosal erosion in patients taking aspirin

	Mantel-Haenszel OR (95% CI)	Adjusted OR (95% CI)
Age, yrs		1.33 (1.04–2.87)*
Creatinine		1.77 (0.62–3.48)
eGFR		8.04 (3.02–22.6)**
History of gastric ulcers	2.74 (1.12–6.72)*	2.41 (1.08–5.01)*
Diabetes mellitus	2.16 (1.23–3.67) **	1.44 (0.87–2.39)
PPIs	0.22 (0.13–0.38)***	0.18 (0.11–0.31)***
SLCO1B1*1b/*1b	2.29 (1.35–3.88)**	2.64 (1.59–4.17)*
SLCO1B1*15/*15	0.10 (0.01–0.84)*	0.26 (0.08–1.31)
SLCO1B1*1a/*15	0.25 (0.09–0.70)**	0.77 (0.36–2.95)

The unadjusted OR and 95% CI were obtained by Mantel–Haenszel statistics, and the adjusted OR and 95% CI were obtained by multiple logistic regression analysis after adjustment for other factors. * *p* < 0.05. ** *p* < 0.01. *** *p* < 0.001

Abbreviations: *eGFR* Estimated glomerular filtration rate, *PPIs* Proton-pump inhibitors

## Discussion

The curative and side effects of aspirin for the prevention and treatment of ischemic cardiovascular diseases are controversial. The ARRIVE trial found that low-risk populations not only benefit from aspirin for the primary prevention of cardiovascular disease but also experience a slightly increased risk of GI bleeding events [7]. The ASCEND study found that the absolute benefit of aspirin in patients with diabetes was largely counterbalanced by the bleeding hazard [8]. Three other articles published in the *New England Journal of Medicine* suggested that aspirin did not reduce the risk of cardiovascular events in elderly patients but was associated with a higher bleeding risk than placebo [9–11]. To our knowledge, our study is the first to evaluate the relationship between *SLCO1B1* polymorphisms affecting the transport of ACEIs or ARBs and aspirin-induced gastric mucosal injury in elderly male Chinese patients. The results showed that the SLCO1B1*1b/*1b diplotype was positively associated with the risk of gastric mucosal erosion in elderly male Chinese patients taking aspirin and an ACEI or ARB concomitantly.

Shiotani et al. considered that the SLCO1B1*1b haplotype and ARBs were both associated with aspirin-induced peptic ulcers in the Japanese population but could not prove the correlation between the SLCO1B1*1b haplotype and ARBs [12]. Additionally, our study emphasized the discovery of early-phase gastric mucosal injury, such as gastric mucosal erosion. The prevention of gastric mucosal erosion is particularly important in the elderly, as it can evolve into a gastric ulcer and even lead to hemorrhage.

Our study also found that the clinical characteristics of increased age, history of gastric ulcers, and no concomitant use of PPIs were risk factors for gastric mucosal erosion. This conclusion is consistent with the finding of another article summarizing the GI bleeding risk factors in aspirin users [13]. Additionally, the level of eGFR decline was found to be associated with gastric mucosal erosion. One possible reason is that aspirin is excreted mainly through the kidneys as a combination of metabolites and free salicylic acid; thus, the degree of gastric mucosal injury may increase as renal function declines. Additionally, the mechanism may be secondary to the local and systemic effects of aspirin [14].

The renin-angiotensin system functions to regulate vasoconstriction and vasodilatation, in which angiotensin II plays a critical role. When the body receives external stress, angiotensin II, the effects of which can be inhibited by ACEI, is released into the blood stream and GI tract tissue, decreasing the gastric submucosal blood supply via vasoconstriction [15]. The other gastroprotective effects of ACEIs and ARBs might include blocking the sympathetic-adrenergic system-mediated inflammatory cascade of tumor necrosis factor α and intracellular adhesion molecule 1, as well as anti apoptosis and extracellular matrix remodeling by downregulating *DDAH/ADMA* and *EGFR/ERK1/2* signaling [16].

ACEIs and ARBs are generally transported into human hepatocytes from portal blood to be metabolized, predominantly via OATP1B1 [17]. Among the mutations identified in the OATP1B1 coding gene (*SLCO1B*), *A388G* and *T521C* occur frequently and are common in the Chinese population (allelic frequencies 73 and 14%, respectively) [18]. There are four haplotypes: SLCO1B1*1a (wild type), SLCO1B1*1b, SLCO1B1*5 and SLCO1B1*15, and the SLCO1B1*1b/*1b (GT/GT) and*1a/*1b (AT/GT) diplotypes have been frequently reported in the Chinese population [19, 20]. The *T521C* single nucleotide polymorphism has been linked to the reduced transport activity of OATP1B1 by affecting the substrate affinity, and the ACEI and ARB concentrations in the blood were reported to be higher in subjects carring the *521C* allele [21]. Although how the protein activity of OATP1B1 is affected by the *A388G* single nucleotide polymorphism is controversial [22, 23], Mwinyi et al. suggested that the activity of OATP1B1 protein transport was significantly higher in *1b/*1b subjects than in *1a/*1a (*AT/AT)* subjects in vivo, implying that the blood drug concentrations may be lower in *1b/*1b subjects [24].

Our study found that the SLCO1B1*1b/*1b diplotype is associated with aspirin-induced gastric mucosal erosion in elderly male Chinese patients taking aspirin and an ACEI or ARB concomitantly. This may be because the reduction in ACEI and ARB concentrations in the blood in individuals with the SLCO1B1*1b/*1b diplotype weakens their protective effect against the gastric mucosa.

The major limitation of the present study was that we did not measure the blood concentrations of ACEIs and ARBs. Thus, the relationship between the change in the degree of drug absorption and gastric mucosal erosion was not proven directly. Another limitation was the use of drugs in elderly patients was complicated, making it challenging to assess all factors that might affect gastric mucosal injury, for example, hormone treatments or anticoagulant drugs.

## Conclusions

The SLCO1B1*1b/*1b diplotype may be a useful genetic predictor for aspirin-induced gastric mucosal erosion in elderly male Chinese patients taking aspirin and an ACEI or ARB concomitantly. Increased age, a history of gastric ulcers, the extent of eGFR decline, and no concomitant use of PPIs are clinical risk factors for gastric mucosal erosion.

## Data Availability

The data that support the findings of this study are available from the corresponding author upon reasonable request.

## Abbreviations

ACEI: Angiotensin-converting enzyme inhibitor
APTT: Activated partial thromboplastin time
ARB: Angiotensin receptor blocker
eGFR: Estimated glomerular filtration rate
GI: Gastrointestinal
HDL-C: High-density lipoprotein cholesterol
LDL-C: Low-density lipoprotein cholesterol
NSAID: Nonsteroidal anti-inflammatory drug
OATP1B1: Organic anion transporting polypeptide 1B1
PLA: People’s liberation army
PPI: Proton-pump inhibitor
SCr: Serum creatinine
SD: Standard deviation
*SLCO1B1*: Solute carrier organic anion transporter 1B1
TC: Total cholesterol
TG: Total triglyceride
